# Risk Factors for Central Lymph Node Metastasis in CN0 Papillary Thyroid Carcinoma: A Systematic Review and Meta-Analysis

**DOI:** 10.1371/journal.pone.0139021

**Published:** 2015-10-02

**Authors:** Wei Sun, Xiabin Lan, Hao Zhang, Wenwu Dong, Zhihong Wang, Liang He, Ting Zhang, Siming Liu

**Affiliations:** Department of Thyroid Surgery, The First Hospital of China Medical University, Shenyang, Liaoning Province, China; Catalan Institute of Oncology, SPAIN

## Abstract

**Background:**

Central lymph node metastasis (CLNM) is common in papillary thyroid carcinoma (PTC). Prophylactic central lymph node dissection (PCLND) for patients with clinically negative central compartment lymph nodes (CN0) remains controversial. The phrase “clinically negative” is used to indicate that patients exhibited no clinical evidence of CLNM by ultrasonography (US) or computerized tomography (CT) preoperatively. In this study, we analyze the risk factors for CLNM in CN0 patients.

**Methods:**

The PUBMED and SCIE databases were systematically searched for works published through January 31, 2015. All of the patients included in this study underwent thyroidectomy+PCLND. Revman 5.3 software was used to analyze the data.

**Results:**

Twenty studies and 9084 patients were included in this meta-analysis. The following variables were associated with an increased risk of CLNM in CN0 patients: age < 45 years (OR = 1.59, 95% CI = 1.42–1.78, p<0.00001), male sex (OR = 1.95, 95% CI = 1.63–2.32, p<0.00001), multifocality (OR = 1.43, 95% CI = 1.22–1.67, p<0.00001), tumor size > 2 cm for PTC patients (OR = 2.98, 95% CI 2.08–4.28, p<0.00001) or tumor size > 0.5 cm for papillary thyroid microcarcinoma (PTMC) patients (OR = 2.30, 95% CI = 1.71–3.09, p<0.00001), location of the primary tumor in the central area and low pole (OR = 1.86, 95% CI = 1.48–2.33, p<0.00001), lymphovascular invasion (OR = 4.35, 95% CI = 2.24–8.46, p<0.0001), extrathyroidal extension (OR = 2.27, 95% CI = 1.76–2.94, p<0.00001), and capsular invasion (OR = 1.72, 95% CI = 1.39–2.41, p<0.00001). PTC (tumor size>1cm) exhibited a higher risk factor associated with CLNM than PTMC (tumor size<1cm) (OR = 2.83, 95% CI = 2.15–3.72, p<0.00001). Bilateral tumors (OR = 1.21, 95% CI = 0.92–1.58, p = 0.17) and lymphocytic thyroiditis (OR = 0.88, 95% CI = 0.71–1.09, p = 0.25) had no association with CLNM in CN0 patients.

**Conclusions:**

Our systematic review identified several clinical features associated with CLNM in CN0 patients, including age, sex, multifocality, size, location, lymphovascular invasion, capsular invasion, and extrathyroidal extension. These factors should guide the application of PCLND in CN0 patients.

## Introduction

Thyroid cancer, the most prevalent endocrine malignancy, accounts for 1% of all malignant neoplasms and 2.7% of all malignant tumors in females, representing the ninth-most common cancer in women[1,2]. Papillary thyroid carcinoma (PTC) is the most common histological subtype of thyroid cancer, comprising 80–85% of all thyroid malignancies[1]. The prognosis of PTC is favorable, with a 10-year survival >91% and 15-year survival >87%[3,4]. Central lymph node metastasis (CLNM) is observed in 20%-90% of patients[5,6]. The significance of lymph node metastasis in PTC patients remains controversial. Previous studies reported that lymph node metastasis may only impact recurrence but not survival[7]. However, recent emerging evidence from a large-scale nested case-control study indicated that lymph node metastasis and incomplete surgical excision are the two primary characteristics associated with higher morbidity[1]. Furthermore, some scholars considered the presence of central lymph nodes to be as important as primary tumors[8].

Prophylactic central lymph node dissection (PCLND) is frequently performed in patients with clinically negative central lymph nodes (CN0). It remains controversial whether elective or prophylactic CLND should be applied in patients with T1 and T2 cancer[9]. The benefits of PCLND for CN0 patients are manifold. First, PCLND can facilitate the diagnosis of an accurate TNM stage and may contribute to the decision to use radioactive iodine (RAI) therapy or thyroid stimulating hormone (TSH) suppressive therapy[10,11]. Second, PCLND may decrease recurrence, increase disease-specific survival, and reduce the Tg levels during postoperative follow-up[7]. In addition, PCLND can help to avoid a reoperation and any associated complications[11,12]. PCLND may be significant for therapy as well as the prognosis of PTC, especially for patients older than 45 years. Because patients older than 45 years exhibit a decreasing capacity to uptake RAI, RAI therapy may be less effective[13]. However, no overwhelming evidence has proven that PCLND definitively improves patient prognosis[14–16]. Although some studies indicate that there is no difference between the outcomes of thyroidectomy and thyroidectomy+PCLND for experienced surgeons[17,18], many authors believe that this statement does not represent the true morbidity rates of patients in whom PCLND is performed. Not all the surgeons are experienced thyroid surgeons, and there is indeed an association between PCLND and postoperative complications[19,20].

Neck ultrasound (US) and contrast enhanced computed tomography (CT) are widely used for preoperative imaging to visualize the CLNM lesions. However, both US and contrast-enhanced CT are not particularly accurate, with low sensitivities of 23%-53.2% and 41%-66.7%, respectively[21,22]. The high incidence of CLNM and low sensitivity of US and CT make it challenging to determine which factors are associated with subclinical CLNM. The phrase “subclinical CLNM” indicates CN0 patients who are proved to have CLNM that is pathologically confirmed intraoperatively or postoperatively. Although many risk factors have been analyzed in many studies for CN0 patients, the results are inconsistent. Thus, we performed a systematic review to assess the clinical features of CLNM in CN0 patients.

## Materials and Methods

Our systematic review was conducted according to the guidelines proposed by the preferred reporting items for systematic reviews and meta-analyses (PRISMA) statement[23]. (S1 PRISMA Checklist.)

### Search strategy

A comprehensive literature research was performed using the PubMed and Web of Science databases for studies published through January 31, 2015 using the key words ((“thyroid cancer or thyroid carcinoma or thyroid neoplasm” AND “papillary”) OR (“PTC”) AND (“central or central compartment or level IV” AND “lymph nodes”)). Two authors (Sun W and Lan XB) performed the selection process independently, and the discrepancies were resolved by discussion.

### Selection criteria

In this systematic review, we included studies that met the following criteria: a) prospective or retrospective original literature; b) English language; c) none of the patients had clinical evidence of CLNM preoperatively; d) all of the patients underwent thyroidectomy plus unilateral or bilateral PCLND, and PTC was pathologically confirmed intraoperatively or postoperatively; and e) completed medical records were available for data extraction. The following exclusion criteria were used to eliminate studies from our meta-analysis: a) patients who had undergone prior head and neck irradiation or oncological surgery; b) reviews, letters to the editor, abstracts or meeting proceedings; and c) patients with a family history of thyroid cancer.

### Data extraction and quality assessment

Two authors abstracted the relevant data from the included articles in accordance with the prepared standardized form. Authors, publication years, countries of study, study design, PTC or papillary thyroid microcarcinoma (PTMC), case number, surgery intervention, 10 possible risk factors and the corresponding numbers of patients were recorded independently. The risk factors included the following: age, sex, multifocality, size, location, lymphovascular invasion, capsular invasion, extrathyroidal extension, bilateral tumors, and lymphocytic thyroiditis. Central lymph nodes were divided into three or four subgroups in some articles: ipsilateral paratracheal, pretracheal, contralateral paratracheal, and prelaryngeal. We included only the data on ipsilateral paratracheal nodes. The Newcastle-Ottawa quality assessment scale was used to assess the quality of the studies.

### Statistical analysis

All of the statistical analyses were performed using Revman software (5.3). The results are presented as odds ratios (ORs) with a 95% confidence interval (CI), and a p value< 0.05 was considered statistically significant, except where otherwise specified. Moreover, heterogeneity was quantified using the Q-test and the I^2^ statistic. When p>0.1 and I^2^<50%, a fixed-effect model was used; otherwise, a random-effects model was applied. Possible publication bias was tested by Begg’s funnel plot.

## Results

**Fig 1:**
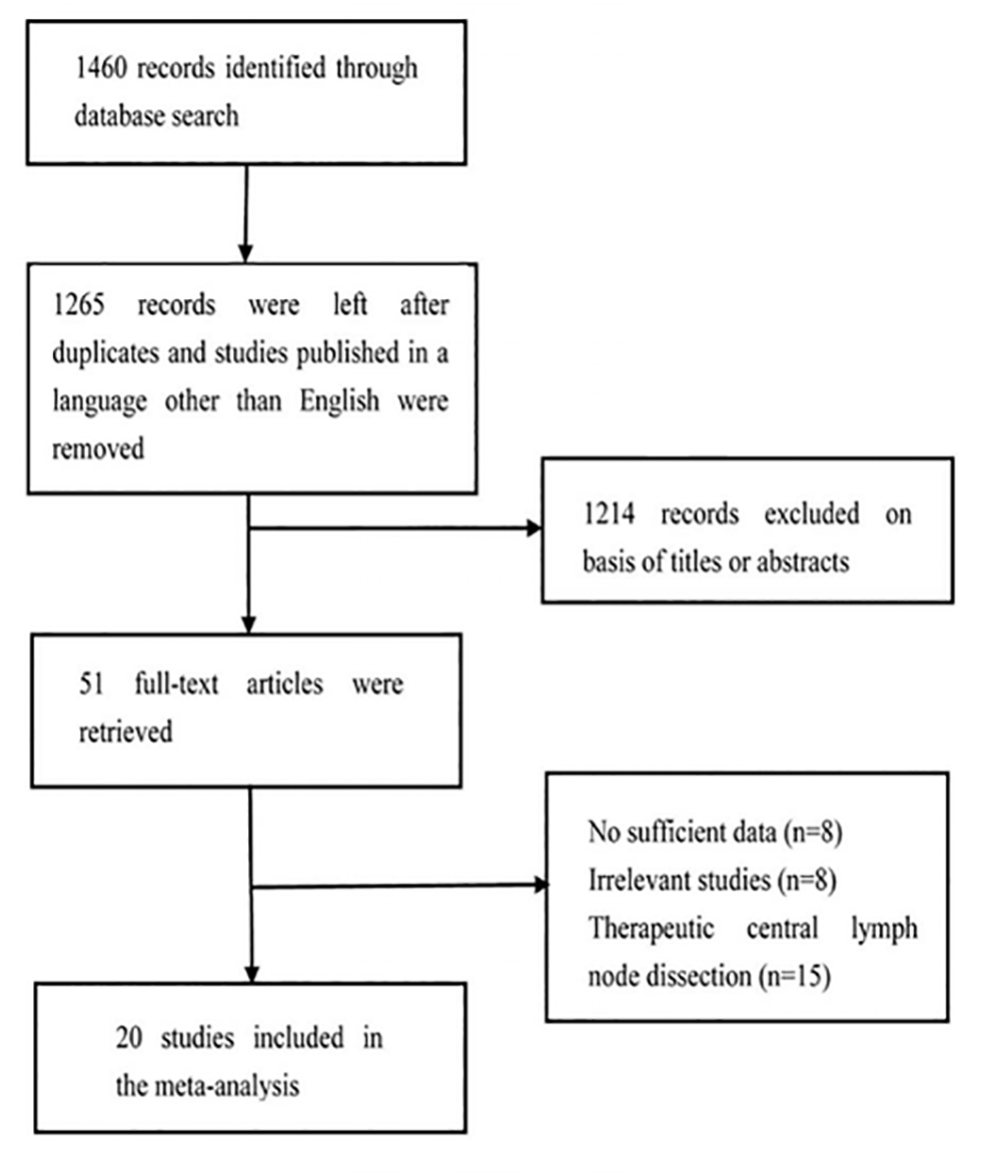
Flow chart of the study selection process.

After screening for studies, 1460 studies were initially considered for inclusion in this meta-analysis. A total of 195 studies were excluded because of duplication and language. Approximately 1214 reviews, case reports, and irrelevant studies were excluded after carefully scanning titles and abstracts. The remaining 51 articles were subjected to an evaluation of the full text. A total of 20 studies and 9084 patients were included in our meta-analysis after applying the inclusion criteria; 3 studies were prospective, and 17 studies were retrospective. The basic characteristics of the papers are summarized in Table 1 and the original data is presented in S1 Table. Begg’s funnel plots are presented in the supporting information section. A flow chart of the selection process for the studies included in the meta-analysis is presented in Fig 1.

**Table 1:** Basic characteristics of included studies.

Author	Year	Country	Study design	PTC/PTMC	Case number	Surgical intervention	Quality assessment
Lim, Y. C[24]	2009	Korea	retrospective study	PTMC	86	TT+bilateral CLND	8
Ying Jie Wu [25]	2013	China	retrospective study	PTC	228	TT +ipsilateral CLND	6
Jong-Lyel Roh [26]	2011	Korea	prospective study	PTC	184	TT+bilateral CLND	8
Q Wang B [27]	2014	China	retrospective study	PTC	188	TT+bilateral CLND or lobectomy plus isthmusectomy+ipsilateral CLND	7
Lie-Hao Jiang [28]	2014	China	retrospective study	PTC	916	TT+bilateral CLND or lobectomy plus isthmusectomy+ipsilateral CLND	7
Wendong Wang [29]	2013	China	retrospective study	PTC	276	TT+bilateral CLND or lobectomy plus isthmusectomy+ipsilateral CLND	7
Yi-Li Zhou [30]	2012	China	Retrospective study	PTMC	122	TT+bilateral CLND	9
Bo-Yeon Kim [31]	2012	Korea	retrospective study	PTMC	160	TT+bilateral CLND	9
Yinlong Yang [32]	2014	China	retrospective study	PTMC	291	TT+bilateral CLND	9
SuSheng Miao[33]	2013	China	prospective study.	PTC	184	TT+bilateral CLND	8
Bon Seok Koo [34]	2009	Korea	prospectively study	PTC	111	TT+bilateral CLND	9
Park J.P. [35]	2014	Korea	retrospective study	PTMC	193	TT+bilateral CLND or lobectomy plus isthmusectomy+ipsilateral CLND	7
Keke Liang [36]	2014	China	retrospective study	PTC	529	TT+bilateral CLND or lobectomy plus isthmusectomy+ipsilateral CLND	7
Yoon Kyoung So [37]	2010	Korea	retrospective study	PTMC	551	TT+bilateral CLND	8
Gilberto Teixeira[38]	2011	Brazil	retrospective study	PTC	72	TT+ipsilateral CLND	7
Yasuhiro Ito [39]	2013	Japan	retrospective study	PTC	3219	TT/NTT+ipsilateral or bilateral CLND	7
Lee K.E. [40]	2012	Korea	retrospective study	PTC	161	TT+bilateral CLND	8
LING-NA MAO [41]	2015	China	retrospective study	PTC	389	TT+ipsilateral or bilateral CLND	7
				PTMC	332	TT+ipsilateral or bilateral CLND	
Mujgan Caliskan [42]	2012	Korea	retrospective study	PTMC	842	TT/NTT+ipsilateral CLND	7
Sang-Hyuk Lee [43]	2008	Korea	retrospective study	PTMC	50	TT+bilateral CLND	9

TT: Total thyroidectomy. NTT: Nearly total thyroidectomy PTMC: papillary thyroid microcarcinoma

### Age

**Fig 2:**
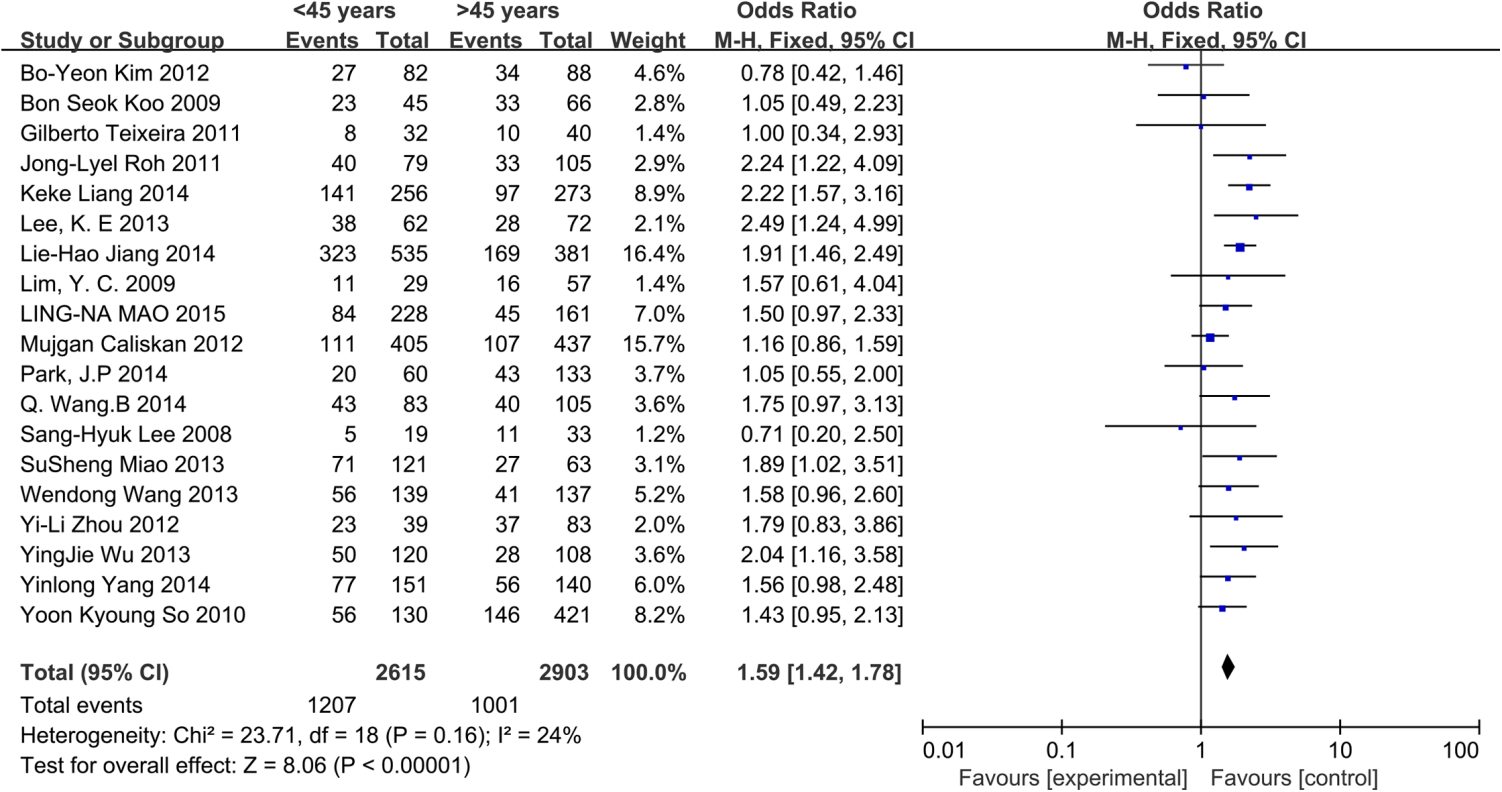
Forest plots of the association between age and CLNM in CN0 patients.

A fixed-effects model was applied in this analysis (p = 0.16, I^2^ = 24%). Among CN0 patients, the rate of CLNM was 46.16% in patients <45 years and 34.48% in the patients >45 years. The results indicate that age <45 years was associated with an increased rate of CLNM in CN0 patients (OR = 1.59, 95% CI = 1.42–1.78, p<0.00001) (Fig 2).

### Sex

**Fig 3:**
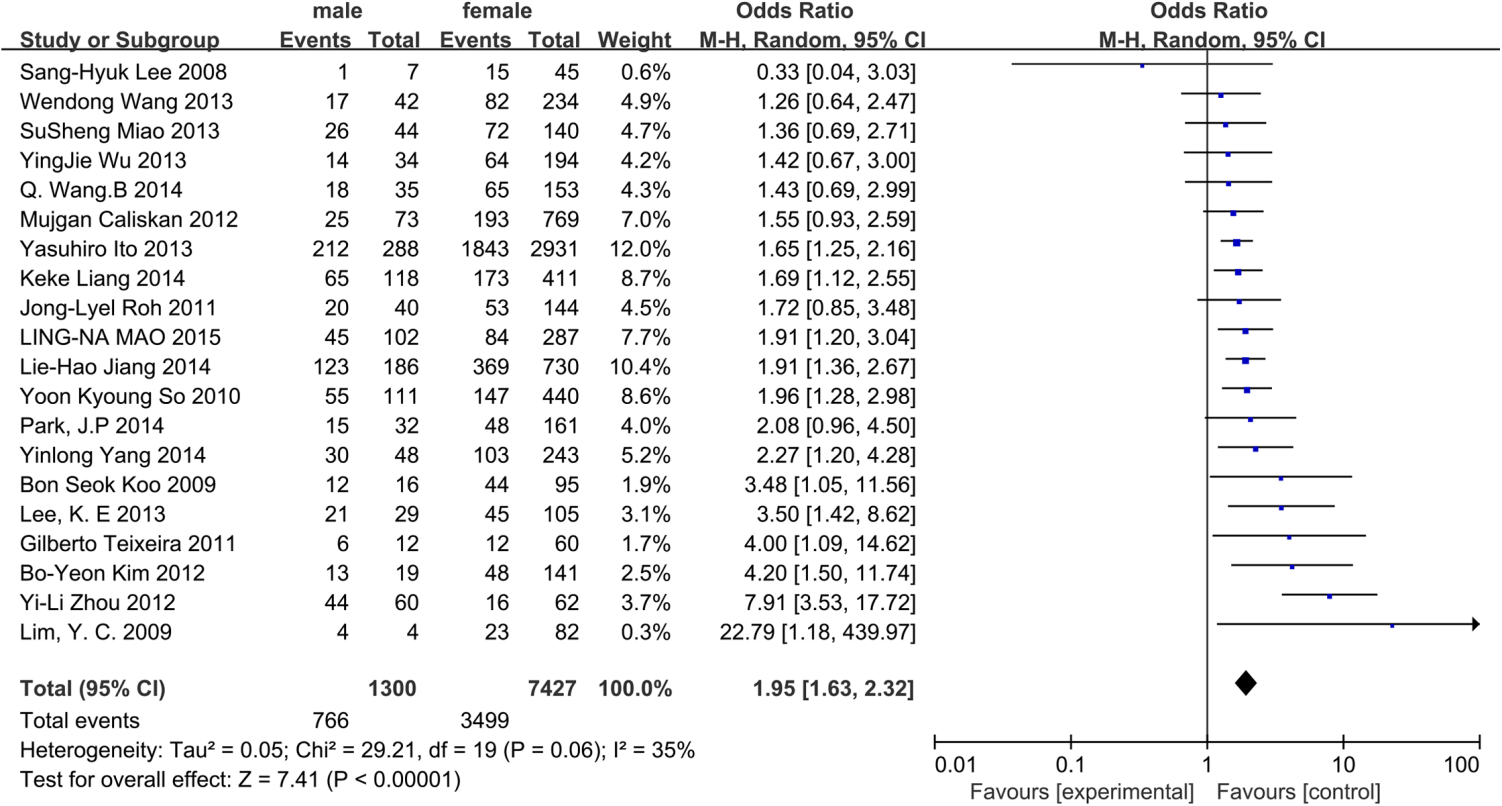
Forest plots of the association between sex and CLNM in CN0 patients.

A random-effects model was adopted to analyze the data (p = 0.06, I^2^ = 35%). The incidence of CLNM was 58.92% in men and 47.11% in women. Male CN0 patients exhibited a significantly higher incidence of CLNM (OR = 1.95, 95% CI = 1.63–2.32, p<0.00001) (Fig 3).

### Multifocality

**Fig 4:**
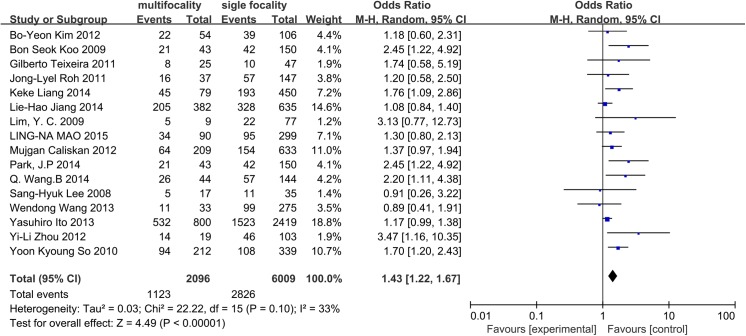
Forest plots of the association between multifocality and CLNM in CN0 patients.

A random-effects model was applied to assess heterogeneity (p = 0.10, I^2^ = 33%). Sixteen studies were included in the analysis of tumor multifocality. We observed a positive correlation between the number of foci and the incidence of CLNM in CN0 patients (OR = 1.43, 95% CI = 1.22–1.67, p<0.0001) (Fig 4).

### Size

**Fig 5:**
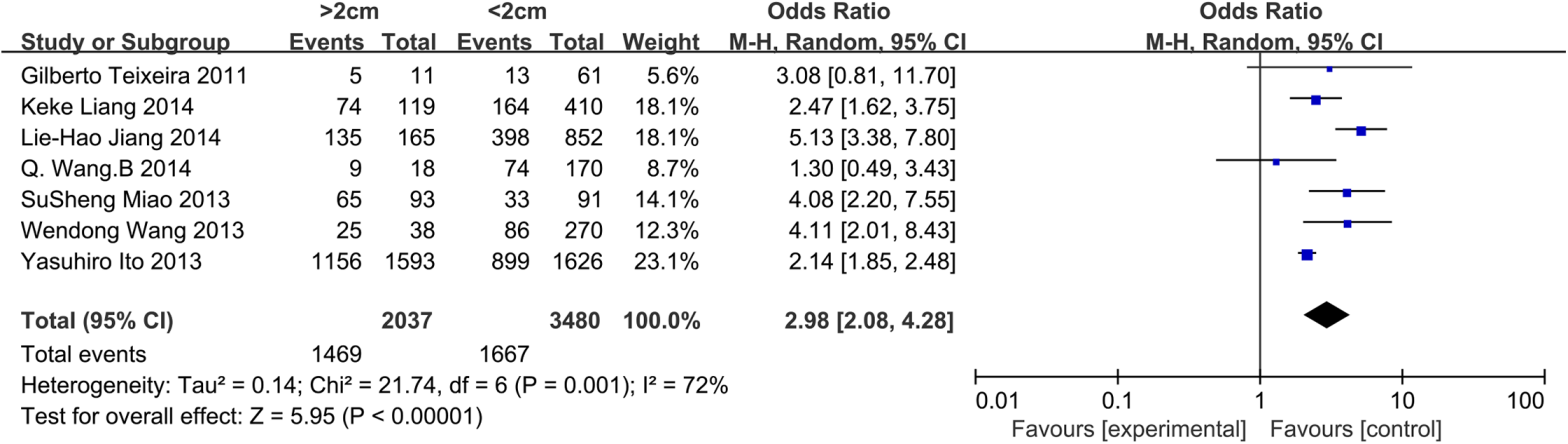
Forest plots of the association between size (PTC) and CLNM in CN0 patients.

**Fig 6:**
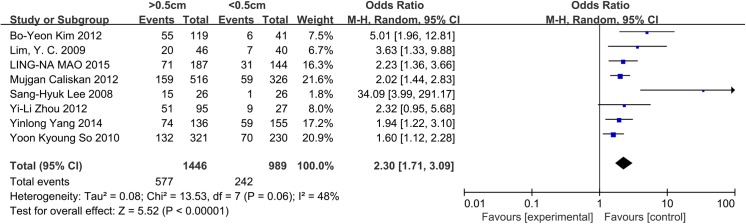
Forest plots of the association between size (PTMC) and CLNM in CN0 patients.

**Fig 7:**
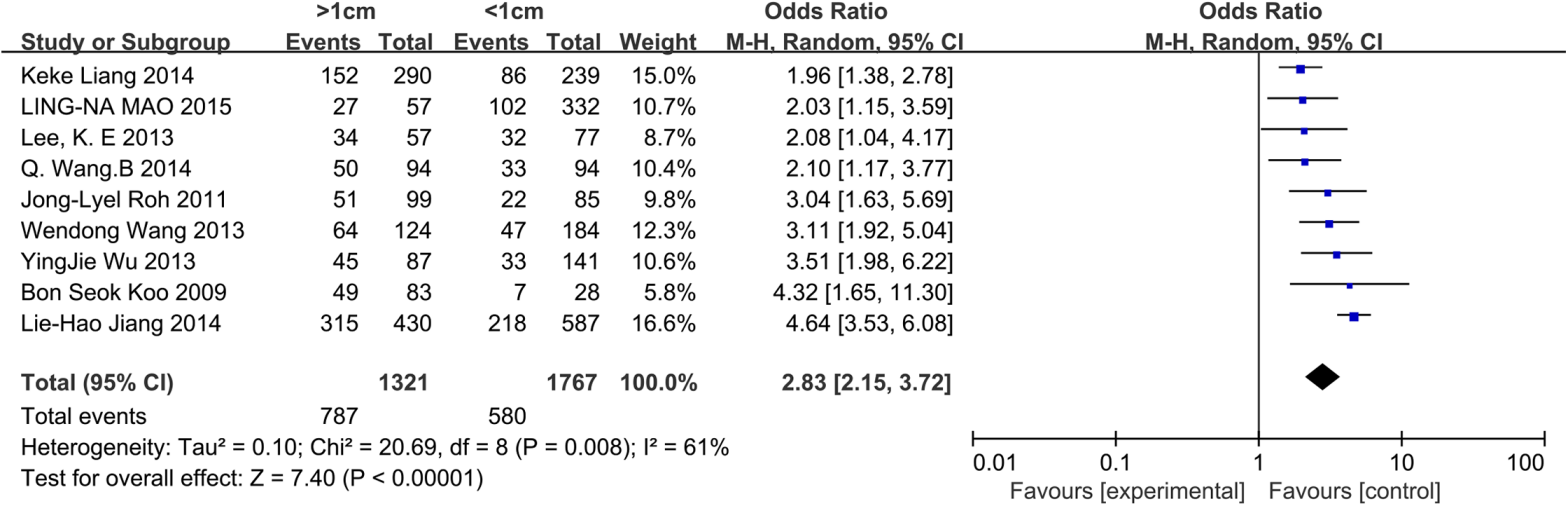
Forest plots of the different prevalence of CLNM between PTC (tumor size>1cm) and PTMC (tumor size<1cm) in CN0 patients.

Seven and eight studies were included in the analysis of the influence of tumor size in patients with PTC and PTMC, respectively. For patients with PTC, a random-effects model was used (p = 0.001, I^2^ = 72%). Tumor size >2 cm was significantly associated with CLNM in CN0 PTC patients (OR = 2.98, 95% CI = 2.08–4.28, p<0.00001). For patients with PTMC, 0.5 cm was established as the cut-off value. A random-effects model was used (p = 0.06, I^2^ = 48%). Tumor size >0.5 cm was identified as a risk factor associated with CLNM in CN0 patients (OR = 2.30, 95% CI = 1.71–3.09, p<0.00001) (Figs 5 and 6). We also analyzed whether the prevalence of CLNM was different between PTC and PTMC. A random-effects model was applied (p = 0.008, I^2^ = 61%). PTC (tumor size>1cm) exhibited a higher risk factor associated with CLNM than PTMC (tumor size<1cm) (OR = 2.83, 95% CI = 2.15–3.72, p<0.00001) (Fig 7).

### Location

**Fig 8:**
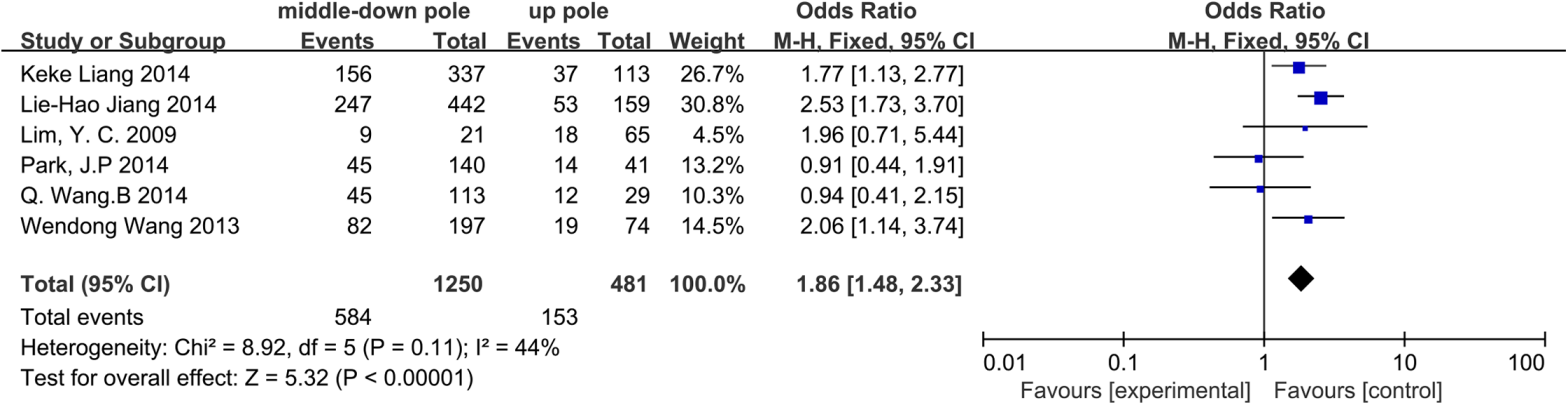
Forest plots of the association between location and CLNM in CN0 patients.

The influence of tumor location on CLNM in CN0 patients was assessed in 6 studies. We divided the thyroid into three areas: upper pole, central area, and lower pole. Approximately 31.81%, 46.77%, and 46.65% located in the upper pole, central area, and lower pole, respectively, exhibited CLNM. When pooling the central area and lower pole for comparison with the upper pole, we observed that tumors located in the central area and lower pole of the thyroid lobe were associated with a high rate of CLNM (OR = 1.86, 95% CI = 1.48–2.33, p<0.00001). A fixed-effects model was used to analyze the data (I^2^ = 44%, p = 0.11) (Fig 8).

### Lymphovascular invasion

**Fig 9:**
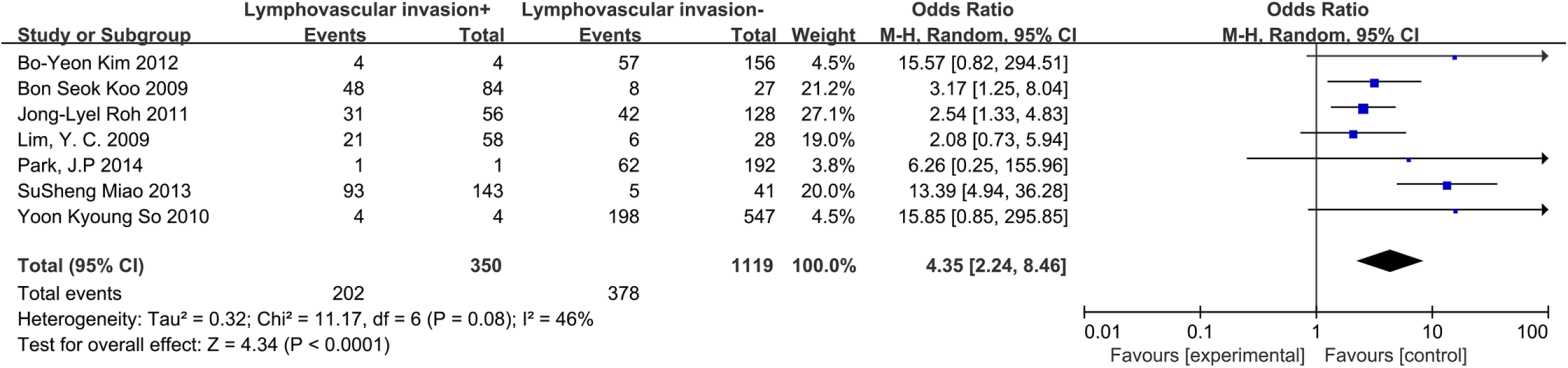
Forest plots of the association between lymphovascular invasion and CLNM in CN0 patients.

A random-effects model was applied to analyze these data (I^2^ = 46%, p = 0.08). Seven included studies were investigated. Lymphovascular invasion exhibited a 4.35-fold increased risk of CLNM in CN0 patients (OR = 4.35, 95% CI = 2.24–8.46, p<0.0001) (Fig 9).

### Capsular invasion

**Fig 10:**
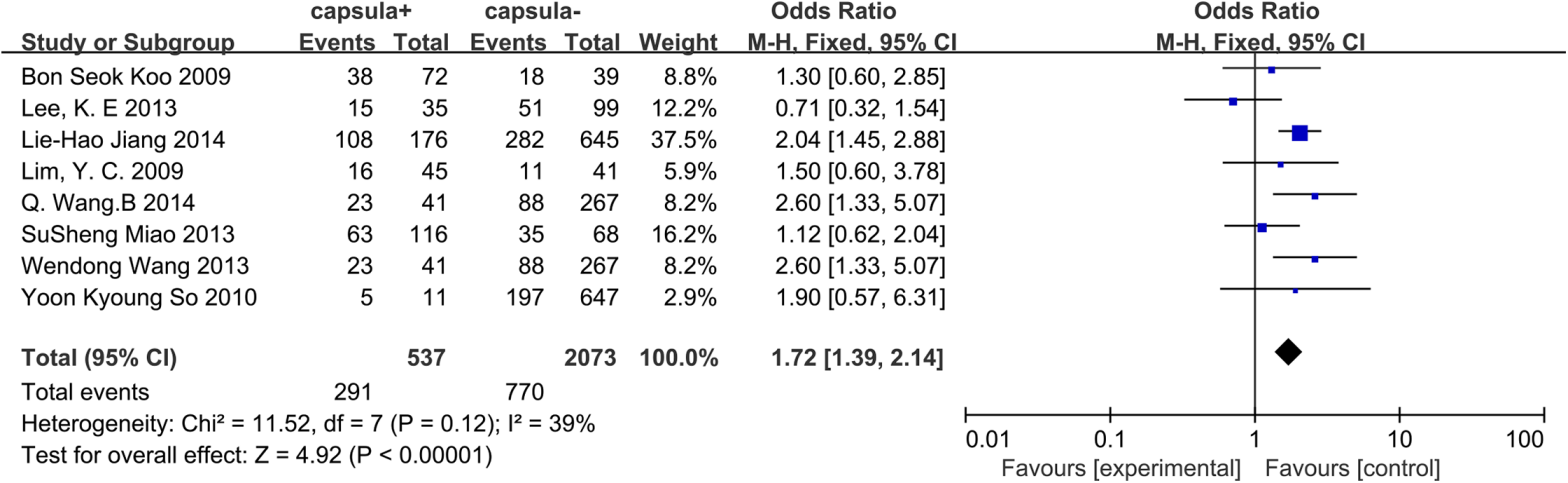
Forest plots of the association between capsular invasion and CLNM in CN0 patients.

A fixed-effects model was applied to our data (p = 0.12, I^2^ = 39%), and 8 studies were included in this analysis. We concluded that for CN0 patients, capsular invasion was associated with CLNM (OR = 1.72, 95% CI = 1.39–2.14, p = 0.001) (Fig 10).

### Extrathyroidal extension

**Fig 11:**
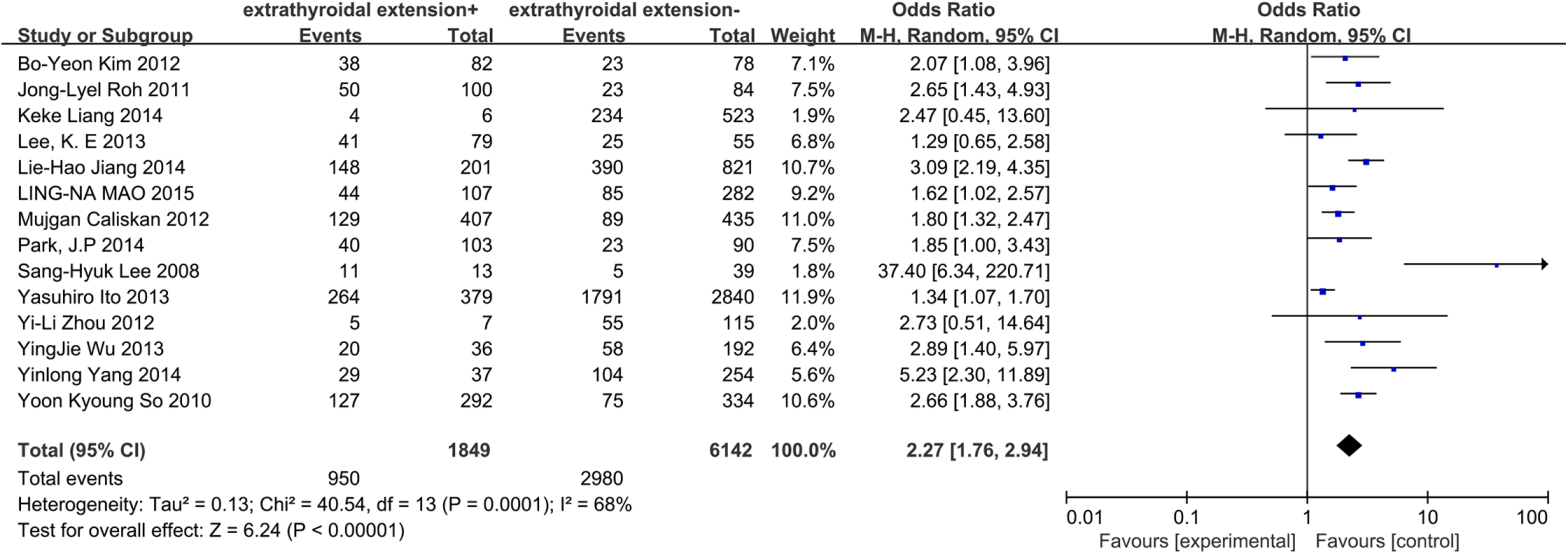
Forest plots of the association between extrathyroidal extension and CLNM in CN0 patients.

A random-effects model determined that the heterogeneity of the data on extrathyroidal extension was significant (p = 0.0001, I^2^ = 68%). A total of 14 papers and 7991 patients were included in this analysis. Extrathyroidal extension exhibited a high propensity to spread to central lymph nodes in CN0 patients (OR = 2.27, 95% CI = 1.76–2.94, p<0.00001) (Fig 11).

### Bilateral tumors

**Fig 12:**
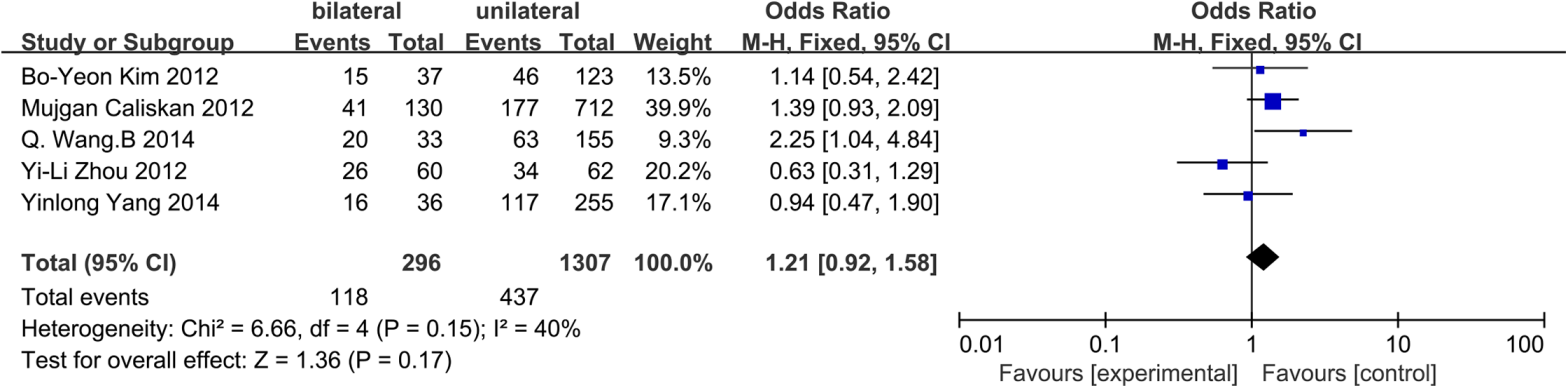
Forest plots of the association between bilateral tumors and CLNM in CN0 patients.

A fixed-effects model was applied due to insignificant heterogeneity (p = 0.15, I^2^ = 40%). Unilateral or bilateral tumors were evaluated in 5 studies. However, neither unilateral tumors nor bilateral tumors were associated with CLNM (OR = 1.21, 95% CI = 0.92–1.58, p = 0.17) (Fig 12).

### Hashimoto’s thyroiditis

**Fig 13:**
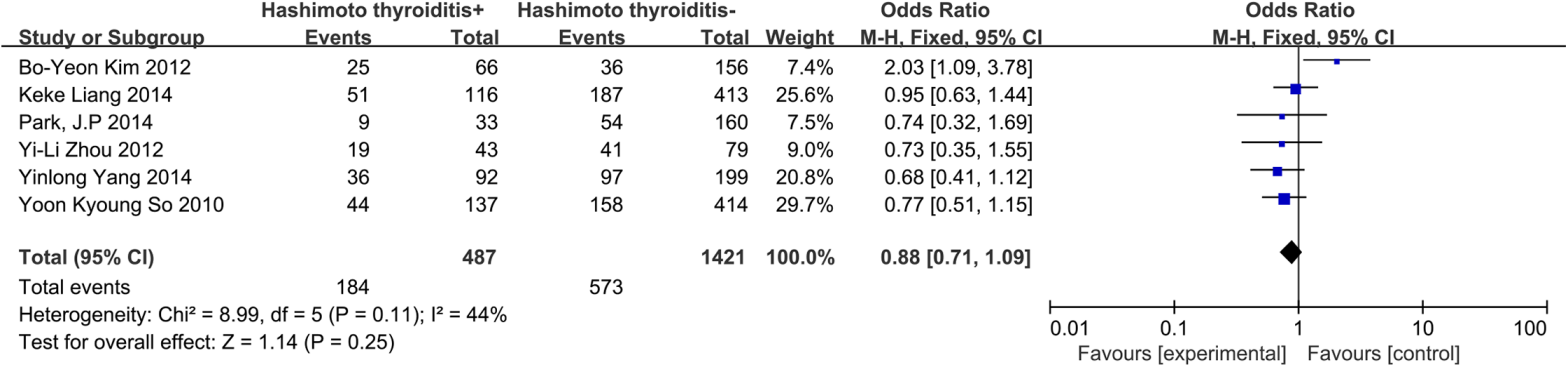
Forest plots of the association between Hashimoto’s thyroiditis and CLNM in CN0 patients.

A fixed-effects model was applied to analyze the data involving Hashimoto’s thyroiditis (I^2^ = 44%, p = 0.11). In our database, a total of 6 studies were included, and CLNM was not associated with the coexistence of chronic Hashimoto’s thyroiditis (OR = 0.88, 95% CI = 0.71–1.09, p = 0.25) (Fig 13).

## Discussion

PTC exhibits low malignancy, relatively good prognosis and a generally positive response to surgery[3,4]. However, PTC also exhibits a high propensity to spread to regional lymph nodes. The central neck compartment is the primary zone of lymphatic involvement in PTC (20%-90% of patients), and CLNM is highly associated with recurrence and overall survival [5,6,44,45]. Patients with papillary thyroid microcarcinoma were also included in our review. PTMC is defined as a tumor that is 10 mm or less along its greatest dimension in accordance with the histologic classification of thyroid tumors by the World Health Organization[31]. The reasons to explore and finally operate on these patients are based on malignant findings from preoperative ultrasonography and fine-needle aspiration biopsy. For PTMC, CLNM appears to affect recurrence but not disease-free survival[15,16,46]. The prevalence of ipsilateral and contralateral CLNM in the included studies in our analysis was 25%-63.83% and 9.24%-30.6%, respectively. The prevalence of ipsilateral CLNM was 25%-49.18% for PTMC (tumor size<1cm) CN0 patients and 47.37%-73.26% for PTC (tumor size>1cm) CN0 patients in our included studies. In the present meta-analysis, PTC (tumor size>1cm) was associated with a higher risk factor than PTMC (tumor size<1cm) in CN0 patients (OR = 2.83, 95% CI = 2.15–3.72, p<0.00001). Preoperative US and contrast-enhanced CT play important roles in screening for CLNM. However, the sensitivities of these techniques are inconsistent, ranging from 23%-53.2% and 41%-66.7% for preoperative US and contrast-enhanced CT, respectively[21,22]. The low sensitivity of US and CT may be due to the following reasons: First, central metastatic lymph nodes are so small that they may not be accurately detected by US and CT. Second, the existence of an intact thyroid gland increases the difficulty of examining posterior lymph nodes around the recurrent laryngeal nerve by US. Third, positive criteria for US and CT in the central neck have not yet been established[47]. In contrast to patients with clinically positive central neck metastasis, there remains controversy concerning how to manage PTC patients with clinically negative CLNM[48].

The leading treatment guidelines are not concordant when compared in relation to their recommendations regarding the application of PCLND. On one hand, the American Thyroid Association (ATA) guidelines recommend that routine PCLND should be performed only in patients with advanced T3 and T4 primary tumors[9]. However, this may not be appropriate, as many T1 or T2 tumors also exhibit CLNM, and different clinicopathologic parameters may play crucial roles in CLNM[43]. The ATA restricts PCLND due to the limitations of its influence on prognosis and because the majority of thyroid surgeries in the United States are performed by surgeons who, according to National Inpatient Sample from 1988 through 2000, perform few such surgeries[49]. Currently, the number of surgeries performed by high-volume surgeons in USA is growing. Cases performed by high-volume surgeons increased from 12% in 1993 through 2000 to 25% in 2001 through 2008, in contrast cases performed by very-low volume surgeons decreased from 51% to 34%[50]. Inexperienced surgeons are associated with a greater rate of complications[51]. However, the Japanese Association of Endocrine Surgeons (JAES) routinely recommends PCLND. This may be attributed to the fact that the use of RAI is limited due to legal restrictions[52]. In conclusion, to more appropriately select patients for PCLND, a careful preoperative evaluation of the risk factors of CLNM in CN0 patients is required.

Many studies have focused on the risk factors of CLND for CN0 patients. However, the results are inconsistent. Age has been reported to be a risk factor in many studies. The cut-off age of 45 years is widely used as a clinical marker for prognosis[5]. Traditionally, patients older than 45 years are associated with poor prognosis and increased recurrence[53]. In our study, we observed that age younger than 45 years is a significant risk factor for CLNM in CN0 patients. Some authors have reported a significant correlation between a higher risk of thyroid cancer and age younger than 45 years[54,55]. Do BA et al. reported that well-differentiated thyroid cancer and lymph node metastasis occurred more often in patients younger than 50 years[56].

Although the incidence of thyroid cancer is higher in women, the rates of malignancy and mortality due to thyroid cancer are higher in men[57,58]. Male sex was identified as a risk factor when patients with thyroid nodules were evaluated, and this factor may be suggestive of thyroid carcinoma[59]. Male patients are also more prone to unhealthy lifestyles and harmful environmental factors, such as smoking and drinking. Several studies have reported that men exhibited a poorer prognosis than women [60,61]. We concluded that male sex is a significant risk factor for CLNM in CN0 patients.

As always, tumor size is an important factor in TNM staging, and large tumors are more prone to be aggressive[62]. In our data, we observed that tumor size >2 cm exhibited a 2.98-fold increased risk of CLNM in CN0 patients. For PTMC, most studies consider 0.5 cm to be the cut-off value. In the present meta-analysis, we observed that tumors larger than 0.5 cm constitute an apparent risk factor. Other studies have reported that tumor sizes smaller than 0.5 cm exhibit no micrometastases or contralateral CLNM in CN0 patients[18,32].

Some studies have reported that multifocal PTC increases the risk of locoregional recurrence as well as lymph node metastasis and distant metastasis[63–65]. For years, multifocal PTC was considered the intraglandular spread of the primary tumor. Thus, multifocal PTC may be more aggressive than unifocal PTC[66]. Ning Qu et al. reported that an increasing number of tumors was associated with a tendency toward more aggressive features and predicted poor prognosis in PTC[43]. Whether multifocality is related to CLNM in CN0 patients remains controversial. In our study, we concluded that multifocality is a risk factor for CLNM.

The association between Hashimoto’s thyroiditis (HT) and PTC was first proposed by Dailey et al. in 1955[67]. It remains controversial whether HT is associated with CLNM in PTC patients. Some studies suggest that the coexistence of HT is not associated with CLNM in PTC or PTMC patients[31,68]. However, another meta-analysis reported that PTCs with coexistent HT were significantly related to the absence of lymph node metastasis[69]. Our data indicated that there was no association between HT and CLNM in CN0 patients. The differences between our findings and previous conclusions may be related to different selection criteria and different study designs.

There are several limitations of this study. First, the included studies were not randomized case-control trials. Second, the types of thyroidectomies performed on the CN0 patients were not consistent and included total thyroidectomy, nearly total thyroidectomy, and lobectomy plus isthmusectomy. Third, we only recorded ipsilateral CLNM from the studies in which level VI was divided into three or four groups. This recording method could have led to some unavoidable bias, as some patients only exhibited contralateral CLNM. Fourth, most of the patients from the included studies were from Asia.

In conclusion, this meta-analysis identified the following significant risk factors for central lymph node metastasis in CN0 patients: age, sex, multifocality, tumor size, tumor location, lymphovascular invasion, capsular invasion, and extrathyroidal extension. Bilateral tumors and Hashimoto’s thyroiditis do not appear to be correlated with CLNM in CN0 patients.

## Supporting Information

S1 PRISMA Checklist PRISMA 2009 Checklist. (DOC)

S1 Fig Begg’s funnel plot of the association between age and CLNM in CN0 patients. (EPS)

S2 Fig Begg’s funnel plot of the association between sex and CLNM in CN0 patients. (EPS)

S3 Fig Begg’s funnel plot of the association between multifocality and CLNM in CN0 patients. (EPS)

S4 Fig Begg’s funnel plot of the association between size (PTC) and CLNM in CN0 patients. (EPS)

S5 Fig Begg’s funnel plot of the association between size (PTMC) and CLNM in CN0 patients. (EPS)

S6 Fig Begg’s funnel plot of the different prevalence of CLNM between PTC (tumor size>1cm) and PTMC (tumor size<1cm) in CN0 patients. (EPS)

S7 Fig Begg’s funnel plot of the association between location and CLNM in CN0 patients. (EPS)

S8 Fig Begg’s funnel plot of the association between lymphovascular invasion and CLNM in CN0 patients. (EPS)

S9 Fig Begg’s funnel plot of the association between capsular invasion and CLNM in CN0 patients. (EPS)

S10 Fig Begg’s funnel plot of the association between extrathyroidal extension and CLNM in CN0 patients. (EPS)

S11 Fig Begg’s funnel plot of the association between bilateral tumors and CLNM in CN0 patients. (EPS)

S12 Fig Begg’s funnel plot of the association between Hashimoto’s thyroiditis and CLNM in CN0 patients. (EPS)

S1 Table Original data of this systematic review and meta-analysis. (XLS)
